# Alpha-1 Antitrypsin Attenuates M1 Microglia-Mediated Neuroinflammation in Retinal Degeneration

**DOI:** 10.3389/fimmu.2018.01202

**Published:** 2018-05-30

**Authors:** Tian Zhou, Zijing Huang, Xiaowei Zhu, Xiaowei Sun, Yan Liu, Bing Cheng, Mei Li, Yizhi Liu, Chang He, Xialin Liu

**Affiliations:** State Key Laboratory of Ophthalmology, Zhongshan Ophthalmic Center, Sun Yat-sen University, Guangzhou, China

**Keywords:** alpha-1 antitrypsin, immunomodulation, microglia polarization, rd1 mice, neuroinflammation

## Abstract

Neurodegenerative diseases are a set of disorders characterized by progressive neuronal death and are associated with microglia-mediated neuroinflammation. Recently, neuroinflammation is proposed as a promising therapeutic target for many neurodegenerative diseases. Alpha-1 antitrypsin (AAT) is recognized as a novel immunomodulatory agent in autoimmune diseases and transplantation, however, its impact on neuroinflammation and neurodegeneration remains unknown. This study aims to explore the effects of AAT on microglia-mediated neuroinflammation and retinal degeneration in rd1 mouse model. We found reduced expression of AAT in rd1 retina, and AAT supplement exhibited certain protective effect on retinal degeneration, presenting with increased amount of photoreceptor nuclei, and amplified wave amplitudes in electroretinogram analysis. Of note, AAT shifted microglia phenotype from pro-inflammatory M1 (CD16/CD32^+^, iNOS^+^) to anti-inflammatory M2 (CD206^+^, Arg1^+^) both *in vivo* and *in vitro*, underscoring the concept of immunomodulation on microglia polarization by AAT during neurodegeneration. Furthermore, AAT suppressed the activation of STAT1, promoted the expression of IRF4 while inhibited IRF8 expression, indicating the involvement of these signaling pathways in AAT immunomodulation. Collectively, our data provided evidence for a novel protective role of AAT through immunomodulation on microglia polarization. Attenuating neuroinflammation by AAT may be beneficial to retard neurodegeneration in rd1 mice.

## Introduction

Neurodegenerative diseases are a heterogeneous group of disorders characterized by progressive loss of specific neurons and association with neuroinflammation. Retinitis pigmentosa (RP), one of the most common inherited retinal degeneration, is a major cause of incurable vision loss (1). It is a highly complex neurodegenerative disease defined by progressive degeneration of photoreceptors or retinal pigment epithelium (RPE) caused by various mutations (2). So far, more than 200 mutant genes have been identified (3, 4) and gene therapy therefore faces the challenge of genetic heterogeneity, which makes mutation-independent approaches highly desirable (5). Drugs that target on the broad biological processes which are common across various mutations in RP may show some efficacy in this complicated disorder (6).

Neuroinflammation is now recognized as a hallmark of many chronic neurodegenerative disorders and attenuating neuroinflammation has been proved to be a potential therapeutic strategy for these diseases (7, 8). It has been reported in RP patients with a substantial number of inflammatory cells found in the vitreous cavity and significantly elevated expression of a variety of pro-inflammatory cytokines and chemokines (9), highlighting that inflammation was implicated in retinal degeneration. Growing evidence suggested that microglia, the tissue-resident immune cells, would initiate immune responses with a chronic inflammation sustained in RP (10). The onset of microglia-mediated inflammation is identified as a common feature of RP (11).

Alpha-1 antitrypsin (AAT), an endogenous serine protease inhibitor for neutrophil elastase (NE), is emerging as a novel immunomodulatory agent to intervene immune diseases and transplantation (12). It is mainly produced by hepatocytes and maintains homeostasis with normal serum levels of 2–5 mg/mL (13). Patients with inherited AAT deficiency are predisposed to the development of lung diseases due to excessive destructive NE (14). And the AAT augmentation is well acknowledged in clinic for the treatment of these individuals (15). Interestingly, it was reported that a patient with RP was identified heterozygous for AAT deficiency (16). It is still unknown if there is a relationship between AAT deficiency and RP, and whether AAT augmentation is suitable for RP patients. Actually, besides its ability for elastase degradation, AAT was recently reported to suppress adverse immune responses under various conditions. For example, AAT application could avert type 1 diabetes and prolong the survival of islet allograft in NOD mice (12, 17). AAT administration reduced the susceptibility of islets to inflammation (18). These findings proposed AAT as a novel alternate for immunosuppression. The microglia-mediated neuroinflammation is an important pathological process in neurodegenerative diseases and immune modulation targeting on microglia is a promising treatment for these diseases. Therefore, we proposed that AAT may have the potential to attenuate microglia-mediated neuroinflammation and retinal degeneration.

In the present study, the impact of AAT immunotherapy on retinal inflammation and neurodegeneration was examined in rd1 mice, a typical mouse model of RP, as well as in cell culture. The results showed that AAT suppressed microglia-mediated neuroinflammtion and protected the degenerative retina. Our data proved that AAT may be beneficial to retinal degeneration by shifting retinal microglia from proinflammatory M1 to anti-inflammatory M2 phenotype.

## Materials and Methods

### Rd1 Mice and AAT Treatment

Rd1 (FVB/N) mice were purchased from Beijing Vital River Laboratory Animal Technology, Co., Beijing, China and were kept in a specific pathogen-free facility in Animal Laboratories of Zhongshan Ophthalmic Center. This study was approved by the animal experimental ethics committee of Zhongshan Ophthalmic Center, Sun Yat-sen University (authorized number: 2014-039). All the experiments were carried out in accordance with the approved guidelines of Animal Care and Use Committee of Zhongshan Ophthalmic Center and the Association Research in Vision and Ophthalmology (ARVO) Statement for the Use of Animals in Ophthalmic and Vision Research. Rd1 mice and C57BL/6J mice at P4, P10, P14, and P20 were sacrificed for detecting AAT expression in retina. For treatment, rd1 mice received intraperitoneal injections of AAT (soluted in PBS 80 mg/kg, Sigma Chemical Co., St. Louis) every other day from P4 to P14. Mice were sacrificed at P16 and eyes were enucleated for further investigation, normal C57 mice of the same age were used as the control.

### Primary Microglia and BV2 Cell Line Culture

Primary microglia were isolated and cultured as described previously (19). In brief, retinae were collected from 3-day-old Sprague–Dawley rats and crashed drastically into single cell suspensions. The cells were then resuspended in DMEM/F-12 medium containing 10% FBS, 100 U/mL penicillin, and 100 mg/mL streptomycin, and were seeded at a density of 1 × 10^6^ cells/mL. The culture medium was changed 24 h later and twice a week thereafter. 2 weeks later, after shaking the flasks at 200 rpm for 1 h, the suspensions were collected and centrifuged at 300 × *g* for 8 min at 4°C. The cells were harvested and identified as microglia using IBA1 staining. The BV2 murine microglial cell line was purchased from Kunming Institute of Zoology, Chinese Academy of Sciences, China. The microglia cells were pre-treated with AAT (1 mg/mL dissolved in PBS, A6105, Sigma) or PBS for 2 h and then stimulated with hydrogen peroxide (200 μM). Eighteen hours later, the cells were harvested for further analysis.

### Electroretinogram (ERG) Recordings

*In vivo* ERG were performed on rd1 mice treated with AAT or PBS (*n* = 9 for each group) and C57 mice of the same age (*n* = 6). After dark adaptation overnight, mice were anesthetized with pentobarbital sodium diluted in saline. The pupils were dilated with 1% tropicamide and 2.5% phenylephrine and the corneas were anesthetized with 0.5% tetracaine HCl eye drops. ERGs were recorded with gold plated wire loop electrodes contacting the cornea surface as the active electrodes. Stainless steel needle electrodes were inserted into the skin near the eye and into the tail serving as reference and ground leads respectively. The mice were exposed to full-field scotopic flashes of 1.3 ms duration presented by a Ganzfeld (Roland Consult, Germany) with the intensities of 3.0 and 10.0 log cd•s/m^2^. For each intensity, five responses were averaged to the luminance of flash stimuli. Amplitudes of the major ERG components (a- and b-wave) were measured (RETIsystem software) using automated and manual modes.

### Imaging Mouse Retina by Optical Coherence Tomography (OCT)

Optical coherence tomography examination with SPECTRALIS-OCT (Heidelberg, Germany) was performed to detect the retinal structure in rd1 mice treated with AAT or PBS (*n* = 9 for each group) and normal C57 mice (*n* = 6). Briefly, the mice were anesthetized with pentobarbital sodium diluted in saline and the pupils were dilated with 1% tropicamide and 2.5% phenylephrine. Each scan was performed for at least two times, with realignment each time. Both the nasal and temporal retinae within 6 × 3 mm^2^ area adjacent to optic nerve disk were scanned, consisted of 25 scan slices to form 3D acquisition. From the 3D imaging, average retinal thickness was measured within a circle area, with 1.5 mm in radius, centering at 3 mm away from the optic nerve disk.

### Hematoxylin & Eosin Staining

Eyes were fixed in formalin overnight, embedded in paraffin, and were cut into 3 µm vertical slices. Sections were washed in deionized water for 5 min and incubated with hematoxylin buffer for 10 min at room temperature. Then these sections were rinsed in deionized water and dipped in 1% eosin solution for 15 s. After rehydrated in alcohol gradients, slices were washed again and mounted. Histological analyses of retinal tissues were observed under microscope (Leica DM4000, Germany). The amount of cell nuclei in outer nuclear layer (ONL) was counted and analyzed. Images were processed and analyzed with Image J software (Public Domain, imagej.nih.gov/ij/).

### Immunofluorescence Staining on Retinal Cryosection and Whole Mounts

Eyes were enucleated and fixed in 4% paraformaldehyde for 60 min. For cryosection, eyes were embedded in O.C.T. compound (Tissue-Tek) with adjustment of the rim of corneal limbus in vertical direction. The frozen sample was then sliced transversely (8 µm) with a cryostat at −20°C and the cross-sections throughout optic nerve were used for staining and analysis. For retinal whole mounts, the retinae were dissected out. Both cryosections and retinal whole mounts were blocked with 0.5% Triton X-100/5% BSA for 2 h at room temperature and were incubated with primary antibodies overnight at 4°C. After washing with PBS, the slices were incubated with secondary antibodies for 1 h and counterstained with DAPI (Invitrogen) for 5 min before mounted. The primary antibodies included anti-AAT antibody (ab166610, Abcam, MA), anti-Brn3a antibody (ab81213, Abcam), anti-GFAP antibody (ab7260, Abcam), anti-CD11b antibody (ab8878, Abcam), anti-CD68 antibody (ab53444, Abcam), anti-IBA1 antibody (019-19471, Wako; ab15690, Abcam), anti-CD16/32 antibody (553141, BD Biosciences), anti-CD206 antibody (AF2535, R&D Systems), anti-iNOS antibody, anti-Arg1 antibody, and anti-Rhodopsin antibody (sc-7271, sc-18355, sc-57432, Santa Cruz, MA, USA). Antibodies used in this experiment were summarized in Table S1 in Supplementary Material. TUNEL staining (*In Situ* Cell Death Detection Kit, Fluorescein; Roche, IN, USA) was performed according to the manufacturer’s instructions. The images were obtained using Zeiss Axiophot fluorescent microscope and LSCM (LSM710, Carl Zeiss).

### Western Blotting

Retinal and cellular protein were harvested and homogenized in lysis buffer (RIPA, Biocolors, Shanghai, China) containing protease and phosphatase inhibitor mini tablets (Thermo Fisher Scientific, No. 88668; USA). The protein concentration was determined by bicinchoninic acid protein assay. Equal amount of protein was used and western blotting was performed as previously described (20). Primary antibodies included anti-AAT antibody (ab166610, Abcam, MA), anti-STAT1 antibody, anti-pSTAT1 antibody (14994S, 7649S, CST), anti-IRF4 antibody (PA5-21144, Thermo Fisher Scientific, USA), anti-IRF8 antibody, anti β-actin antibody (ab28696, ab28696, Abcam, MA, USA), anti-iNOS antibody, and anti-Arg1 antibody (sc-7271, sc-18355, Santa Cruz, CA, USA). The gray intensity of protein blots was measured using Image J software (US National Institutes of Health).

### Quantitative PCR Analysis

The mRNA levels of AAT were detected by real time PCR. The total RNA of retinae were extracted with TRIzol (Invitrogen) and converted into first-strand cDNA using random hexamer primers and the Reverse Transcriptase Superscript II Kit (Invitrogen) according to the manufacturer’s instructions. Real-time PCR was performed in a total volume of 20 µL containing 2 µL of cDNA, 10 µL of 2 × SYBR Premix Ex Taq, 7 µL ddH_2_O, and 10 µmol/L of the primer pairs. The sequence of the used primers was: AAT forward: 5′-TCCCATGAGATCGCTACAAAC-3′; reverse: 5′-TGATAATGGTTCTTGGCCTCT-3′; GAPDH forward: 5′-GCCAAGGCTGTGGGCAAGGT-3′; reverse: 5′-TCTCCAGGCGGCACGTCAGA-3′. The PCR amplification protocols consisted of 95°C for 30 s and up to 40 cycles of 95°C for 5 s and 60°C for 34 s according to the manufacturer’s instructions.

### Statistics

Statistical analysis was performed using GraphPad Prism (GraphPad Software, Version 6.0, La Jolla, CA, USA). For immunofluorescence on retinal whole mounts, three images were captured in the center, mid-periphery, and periphery areas of each retina respectively. Six retinae from six mice were used in each group for analysis. Representative images were shown in the according Figures. For immunofluorescence, Hematoxylin & eosin, and TUNEL staining on retinal section, at least three sections of each retina and three retinae of each group were used. In every section, three images were captured in the center, mid-periphery, and periphery area, respectively. In experiments including qPCR and western blot, samples were collected from three retinae of three individual mice. All *in vitro* experiments were performed in triplicate and repeated independently for at least three times. Unpaired student *t*-test was used to compare the means between two groups. Data were presented as mean ± SEM and *P* values <0.05 were considered statistically significant.

## Results

### The Expression of AAT Was Reduced in the Degenerative Retinae of rd1 Mice

**Figure 1:**
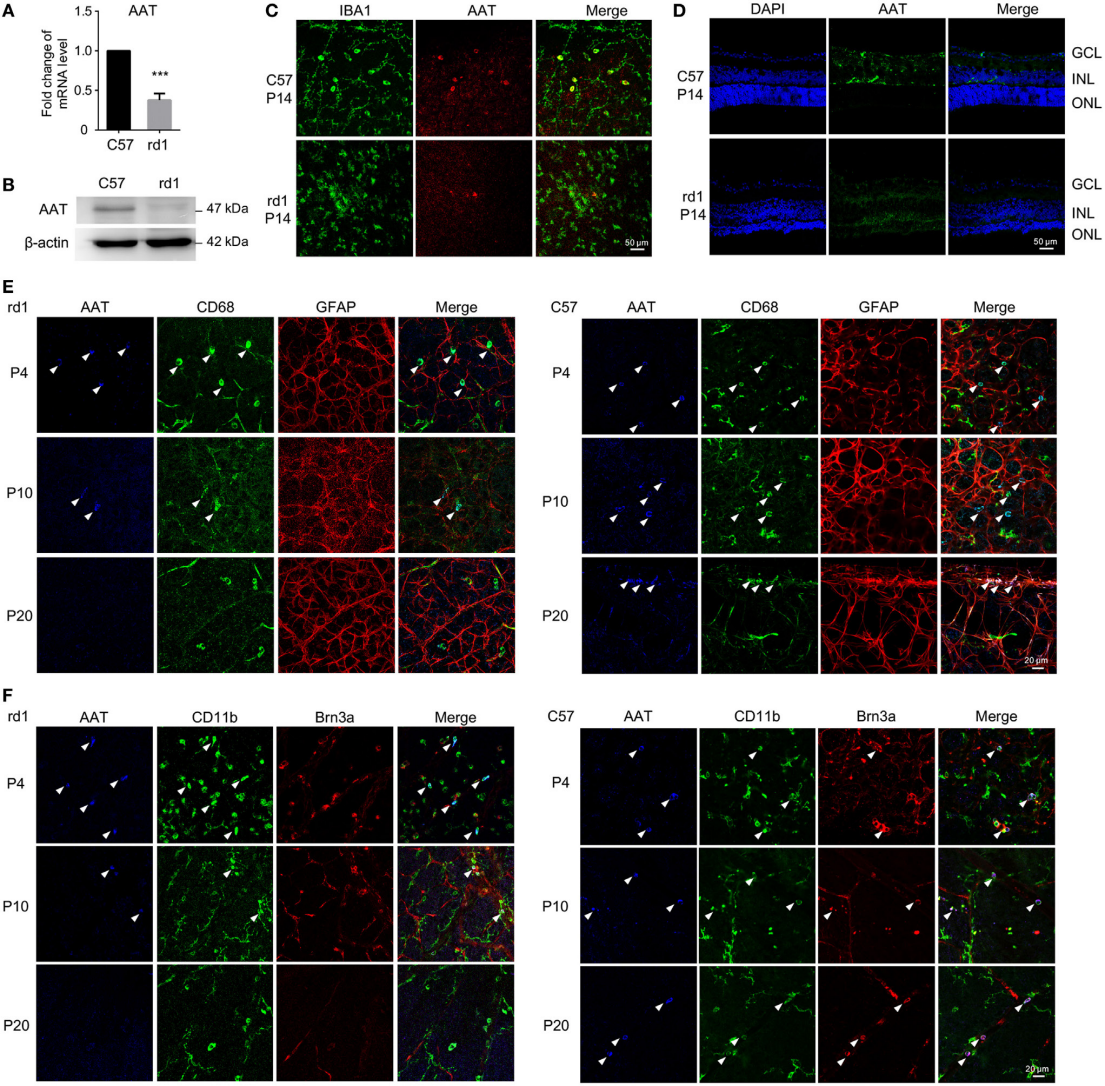
Alpha-1 antitrypsin (AAT) expression decreased in rd1 mice. **(A,B)** Real time PCR **(A)** and western blot **(B)** results revealed that the expression of AAT decreased in retina from rd1 mice compared with C57 controls at P14. ***p < 0.001 (two-tailed unpaired *t*-test). **(C)**. There were some cells co-staining with AAT (red) and IBA1 (green) in C57-retina, whereas lack of AAT was observed in the retina from rd1 mice at P14. **(D)** Immunofluorescence on retinal section showed that AAT was mainly distributed in the inner nuclear layer in C57 mice, while the rd1 mice lacked AAT expression. **(E,F)** In retinal whole mounts, expression of AAT was decreased as the disease progressed from P4 to P20 in rd1 mice, whereas C57 mice presented stable expression of AAT during the same time. Of note, AAT was co-labeled mostly with CD68 **(E)** and CD11b **(F)**, also markers for microglia in the retina, slightly with Brn3a^+^ retinal ganglion cell **(F)**, but not with GFAP^+^ astrocytes/Müller cells **(E)**. Scare bar, 50 µm.

Although AAT is mainly produced by hepatocytes, it could also derive from macrophages, monocytes, and other cells (21, 22). We first detected the expression level of endogenic AAT in rd1 mice retinae. Strikingly, the RNA (Figure 1A) and protein (Figure 1B) levels of AAT were reduced significantly in rd1 retina compared with the C57 controls at P14 when photoreceptors experienced severe loss (23, 24). Furthermore, the high expression of AAT in C57-retina was localized mainly on IBA1^+^ microglia, whereas low level was detected on rd1-microglia at P14 (Figure 1C). The immunostaining on retinal cryosection showed that AAT was mostly distributed along the INL (Figure 1D). To further depict temporal expression of AAT on other retinal cell types, we performed co-staining of AAT and markers for several retinal cell types at different time-points. Strikingly, decreased expression of AAT was observed in rd1 retina as the disease progressed from P4 to P20 and its expression also reduced markedly compared with the same age C57 controls, whereas both mice presented similar levels of AAT at P4 (a few days before the onset of rod photoreceptor degeneration at P9 in rd1) (Figures 1E,F). Of note, AAT co-labeled mostly with CD68 and CD11b, markers for microglia in the retina, while slightly with Brn3a^+^ retinal ganglion cell (RGC), but not with GFAP^+^ astrocytes/Mullers (Figures 1E,F), indicating that microglia was possibly the predominant cell type expressing AAT in the retina.

### AAT Supplement Attenuated the Degenerative Retina of rd1 Mice

**Figure 2:**
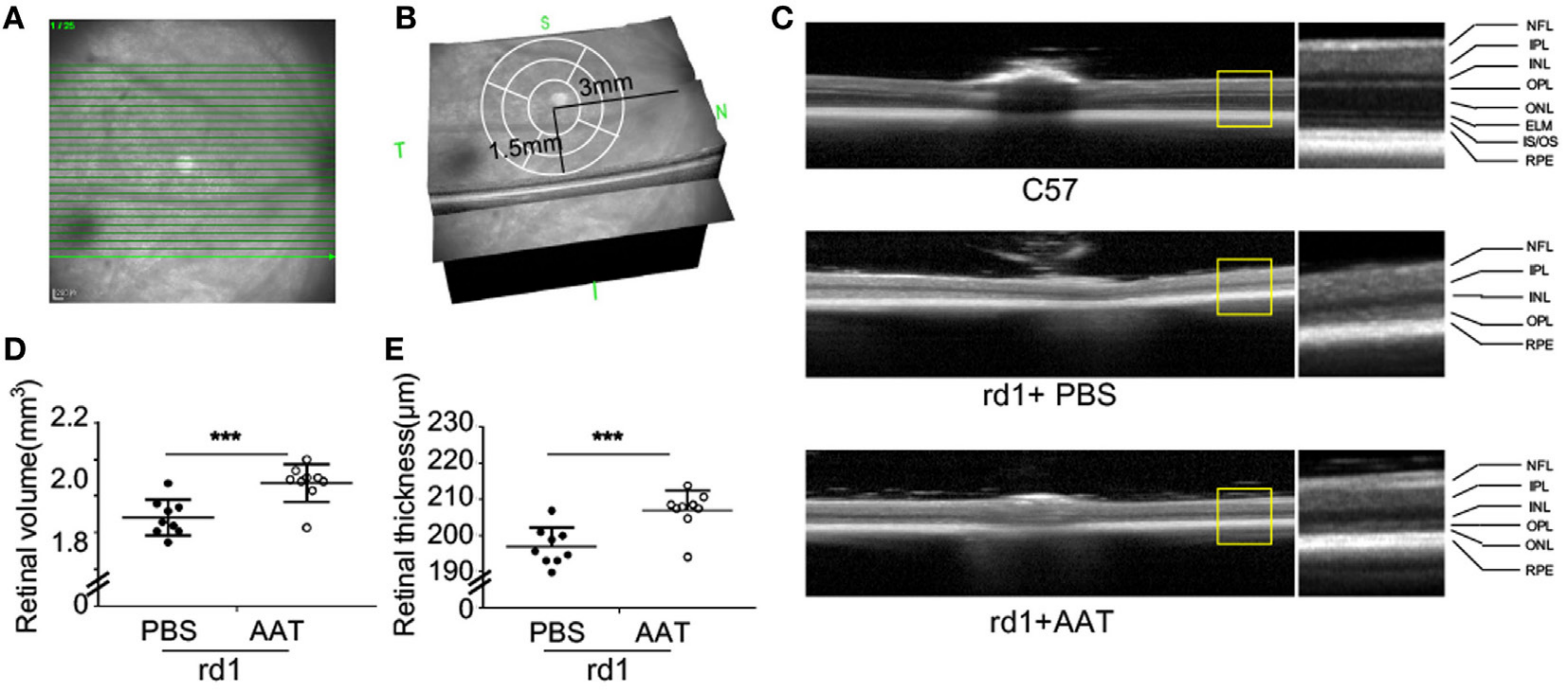
Alpha-1 antitrypsin (AAT) reduced retinal degeneration in rd1 mice. **(A)** The scanning model used in the study. Twenty-five linear scans within 6 × 3 mm^2^ area adjacent to optic nerve disk were obtained at nasal and temporal retinae. **(B)** The average retinal thickness was measured in circle area with a radius of 1.5 mm, centering at 3 mm away from optic nerve disk. **(C)** The retinal structure was well-organized with multiple layers in the C57 mice (*n* = 6), while the layers of ONL, ELM, and IS/OS junction were poorly visible in the PBS-treated rd1 mice at P16. However, the AAT-treated rd1 displayed a visible low-reflecting ONL layer. Abbreviations: NFL, nerve fiber layer; IPL, inner plexiform layer; INL, inner nuclear layer; OPL, outer plexiform layer; ONL, outer nuclear layer; ELM, external limiting membrane; IS/OS, inner segment/outer segment; RPE, retinal pigment epithelium. The retinal volume **(D)** and thickness **(E)** were increased in the AAT-treated rd1 mice compared to PBS-treated ones. *n* = 9 mice for each group. ***p < 0.001 (two-tailed unpaired *t*-test).

**Figure 3:**
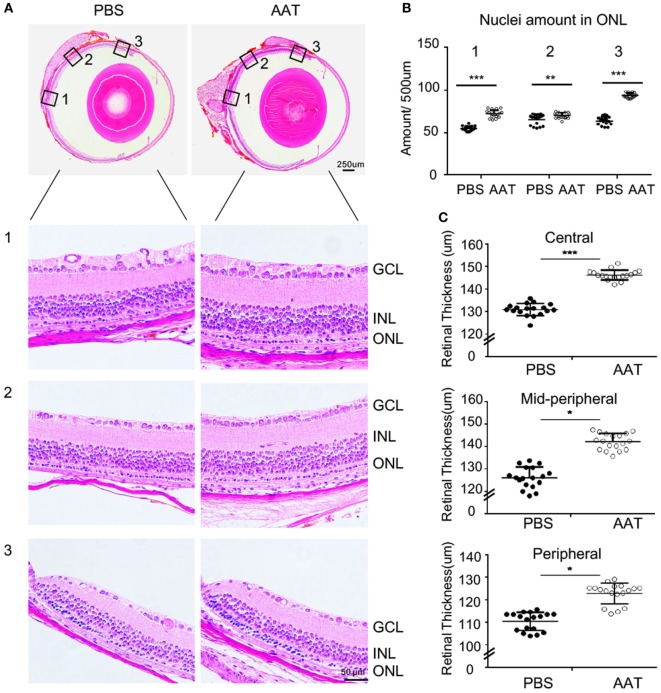
Alpha-1 antitrypsin (AAT) alleviated the decrement of retinal thickness in rd1 mice. **(A)** Panoramic view of the central [Location 1], mid-peripheral [Location 2], and peripheral [Location 3] areas in the retinal paraffin sections by H&E staining. The representative images showed denser nuclei in the outer nuclear layer (ONL) and thicker retinal thickness of the rd1 mice treated with AAT compared to those with PBS treatment at P16. Scare bar, 50 µm. **(B)** The amounts of cellular nuclei in ONL at indicated locations were elevated significantly after AAT treatment in comparison with PBS controls. **(C)** The total thicknesses of retina were increased in the center, mid-periphery, and periphery areas in AAT-treated rd1 mice, compared with those in PBS-treated controls. Six sections of each retina and three retina of each group were used for analysis. *p < 0.05, **p < 0.01, ***p < 0.001 (two-tailed unpaired *t*-test).

Given the decreased level of AAT in rd1 mice, we proposed that AAT supplement might be an alternative intervention for retinal degeneration. To test the possibility of this hypothesis, we injected 80 mg/kg AAT intraperitoneally in rd1 mice every other day from P4 to P14, and evaluated the retinal structure and thickness using SD-OCT and histologic analysis at P16. Twenty-five linear scans within 6 × 3 mm^2^ area adjacent to optic nerve disk were obtained at nasal and temporal retinae (Figure 2A). As depicted in the 3D imaging of Figure 2B, the average retinal thickness was measured within a circle area, with 1.5 mm in radius, centering at 3 mm away from optic nerve disk. As expected, C57 mice presented normal retinal structures with multiple layers, including the nerve fiber layer, inner plexiform layer, inner nuclear layer, outer plexiform layer, ONL, external limiting membrane (ELM), inner segment/outer segment (IS/OS), and RPE. However, these retinal layers were hard to distinguish the PBS-treated rd1 mice with poorly visible ONL, ELM, and IS/OS junctions. Of note, the rd1 mice exhibited a low-reflecting ONL layer after AAT supplement (Figure 2C). The retinal volume and thickness were calculated and mild increment could be observed in rd1 mice after AAT supplement (Figures 2D,E). Histologic analysis was also performed and representative sections were shown in Figure 3A. A thicker retina was observed in AAT-treated retinae at P16. Despite the retinal thickness was slightly elevated in the rd1 mice treated with AAT compared to those under PBS treatment, the increased amount of photoreceptor nuclei in the ONL layer was more significant (Figures 3B,C), indicating the pronounced loss of photoreceptors in rd1 mice was ameliorated by AAT.

### AAT Supplement Improved Retinal Function From Decrement in rd1 Mice at P16

**Figure 4:**
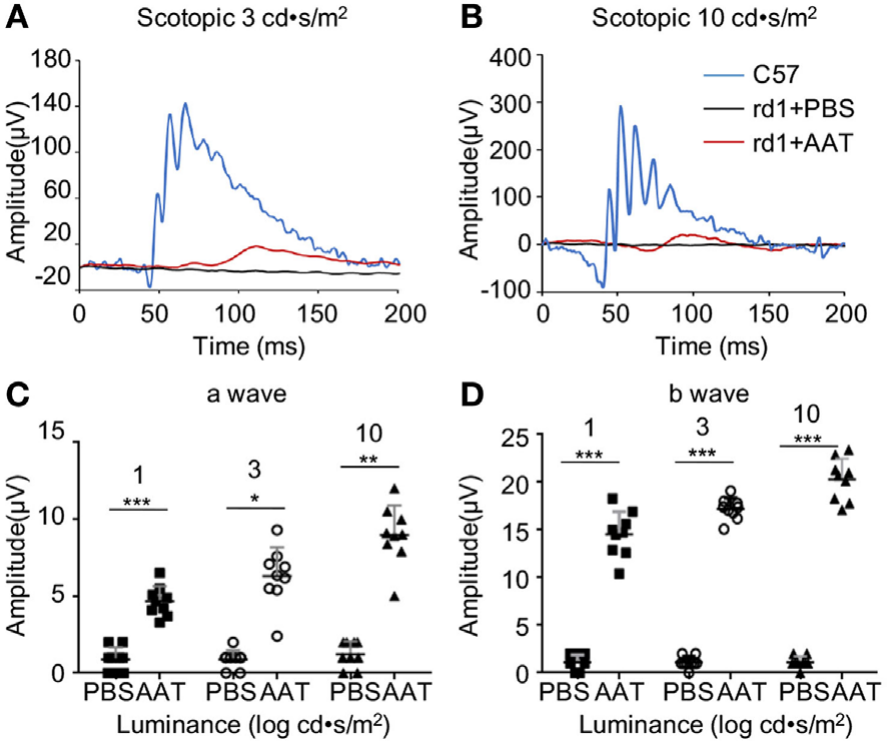
Alpha-1 antitrypsin (AAT) supplement protected retinal function in rd1 mice. **(A,B)** Retinal function was measured at P16 by electroretinogram using single-flash recordings at light intensities of 3.0 and 10.0 log cd•s/m^2^, respectively. C57 mice presented with typical a- and b-wave responses (*n* = 6), while the PBS-treated rd1 mice showed nearly undetectable amplitude of a- or b-waves under a variety of scotopic testing conditions. **(C,D)** AAT treatment induced mild increase in a-wave but significant elevation of b-wave amplitudes in the rd1 mice. *n* = 9 mice for each group. *p < 0.05, **p < 0.01, ***p < 0.001 (two-tailed unpaired *t*-test).

Next, the retinal function was accessed by ERG at P16. The C57 control mice displayed typical a- and b-wave responses, whereas the PBS-treated rd1 mice exhibited virtually flat signal recordings under a variety of scotopic testing conditions. After AAT supplement, recognizable a- and b-waves were recovered, though the ERG responses were still diminished in comparison to C57 mice (Figure 4). Average amplitudes of a- and b-wave for each group of rd1 mice were analyzed. Both a- and b-wave amplitudes were found to be protected with increased amplitudes in the AAT-treated ones, confirming the extinct ERG responses in rd1 mice could be recovered after AAT supplement.

### Inherited Photoreceptor Degeneration Was Protected by AAT

**Figure 5:**
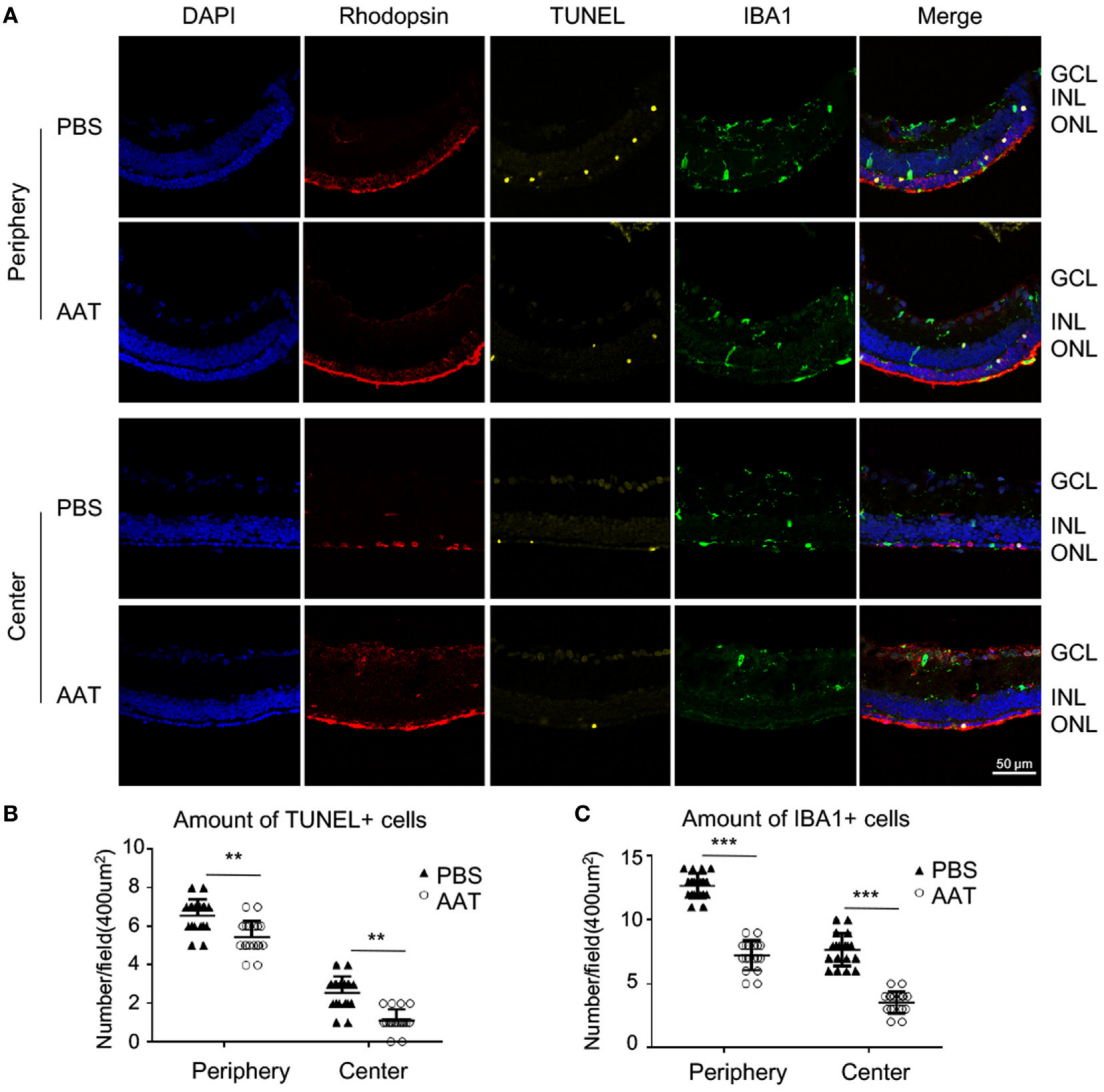
Protective effect of alpha-1 antitrypsin (AAT) on degenerative rods. **(A)** The representative images of immunostaining on retinal sections revealed that rhodopsin-positive rod photoreceptors were scattered in the PBS-treated rd1 mice, but widely distributed and well-organized in the outer nuclear layer after AAT treatment. The TUNEL^+^ apoptotic cells decreased in AAT supplement group, whereas the amount of IBA1^+^ microglia also decreased comparing with the PBS-treated ones. The location “Center” refers to the panoramic view area 1 in Figure 3A, and “periphery” refers to the area 3. Scare bar, 50 µm. Statistical analysis of the TUNEL^+^ **(B)** and IBA1^+^ cells **(C)** revealed that all their amounts significantly decreased either in the center or periphery. Six sections of each retina and three retina of each group were used for analysis. **p < 0.01, ***p < 0.001 (two-tailed unpaired *t*-test).

To further explore whether the AAT preserved the degenerative photoreceptors, rhodopsin and TUNEL staining was performed on retinal sections. More rhodopsin-positive rod photoreceptors were identified in the ONL of AAT-treated rd1 mice compared with the PBS control (Figure 5A). TUNEL-positive cells were evident in the ONL of PBS-treated rd1 mice at P16, while scarce TUNEL-positive cells were observed in the AAT-treated ones (Figures 5A,B), indicating the protective effect of AAT on the apoptotic rods.

### AAT Suppressed Pro-Inflammatory M1 Microglial Polarization During Retinal Degeneration

**Figure 6:**
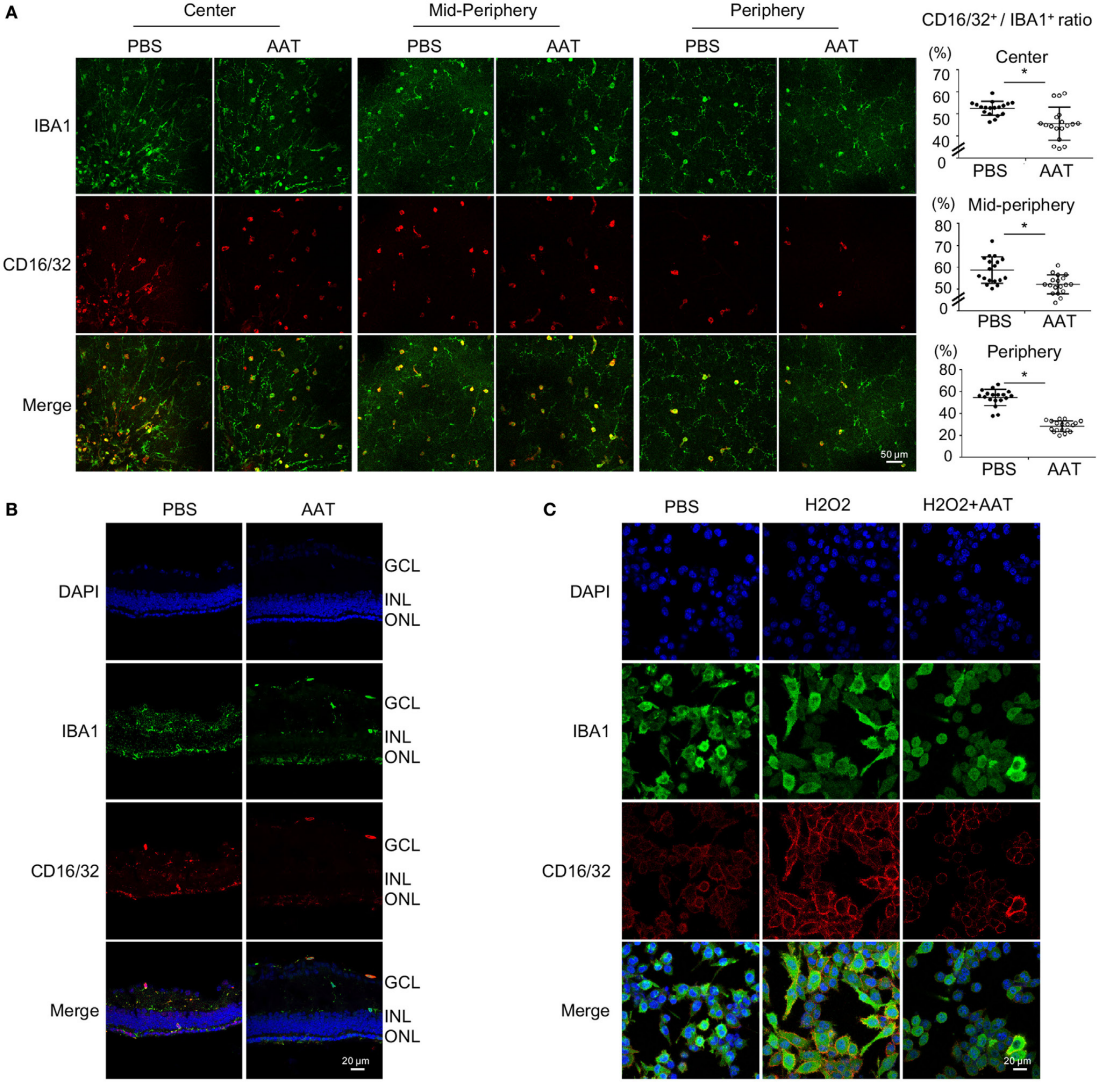
Alpha-1 antitrypsin (AAT) suppressed pro-inflammatory M1 microglial polarization during retinal degeneration. **(A)**. In retinal whole mounts, the amount of CD16/32^+^IBA1^+^ M1 microglia in the central, mid-peripheral, and peripheral retina decreased significantly after AAT supplement. Scare bar, 50 µm. Depicted is mean ± SEM of three fields/eyes from six eyes. *p < 0.05 (two-tailed unpaired *t*-test). **(B)** In the retinal section, CD16/32^+^IBA1^+^ microglia prominently accumulated in the outer nuclear layer in PBS treatment group whereas the amount of CD16/32^+^IBA1^+^ pro-inflammatory microglia significantly decreased in the AAT-treated group. Scare bar, 20 µm. **(C)** In the cultured microglia under oxidative stress by hydrogen peroxide stimulation, AAT supplement significantly suppressed the pro-inflammatory M1 phenotype of microglia, presenting with less CD16/32^+^ cells co-stained with IBA1^+^ cells. Scare bar, 20 µm.

Microglia was recognized as the main immune cell in retina and its activation contributed to neuroinflammation during retinal degeneration (10, 25). In this study, we noticed decreased number of IBA1^+^ positive microglia in the AAT-treated rd1 retina, especially in the degenerative photoreceptor layer of ONL (Figures 5A,C). Since the activated microglia displayed different phenotypes at various conditions, we detected the pro-inflammatory M1 phenotype at the setting of degenerative retina in rd1 mice. CD16/32 is widely used as a classic marker for M1 microglia. As demonstrated in Figure 6A, AAT supplement obviously decreased the ratio of CD16/32^+^IBA1^+^ M1 microglia compared with that of the PBS-treated ones, prominently at the peripheral retina. In retinal sections, CD16/32^+^IBA1^+^ microglia accumulated in the ONL of PBS-treated mice, whereas a lack of this pro-inflammatory microglia was observed in the AAT group (Figure 6B). In cultured BV2 microglia under oxidative stress by hydrogen peroxide stimulation, which mimic the general injury in the rd1 retina, AAT supplement also downregulated the expression of pro-inflammatory marker CD16/32^+^ on microglia (Figure 6C). These results suggested that AAT suppressed the pro-inflammatory M1 microglia during retinal degeneration.

### Microglia Skewed Toward Anti-Inflammatory M2 Phenotype in the Presence of AAT Supplement

**Figure 7:**
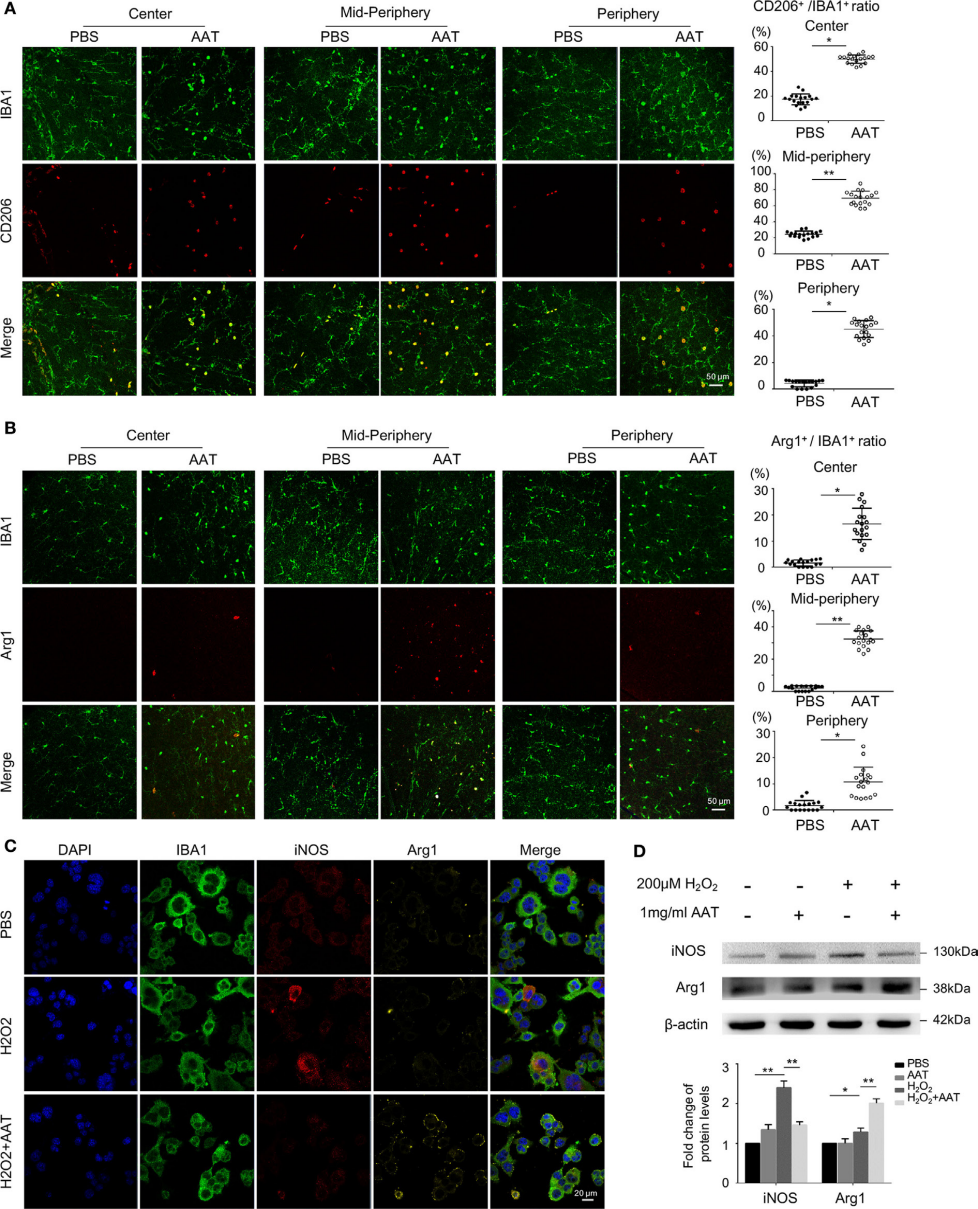
Microglia skewed toward anti-inflammatory M2 phenotype in the presence of alpha-1 antitrypsin (AAT) supplement. **(A)** In the retinal whole mounts, the amount of CD206^+^IBA1^+^ microglia significantly increased in AAT-treated group compared with the PBS-treated mice, particularly in the central and mid-peripheral retina. **(B)** Arg1^+^ cells, another M2 microglia marker, were absent in the PBS-treated rd1 retina, while after AAT supplement, these M2 microglia appeared and most concentrated in the mid-peripheral areas. Scare bar, 50 µm. Depicted is mean ± SEM of three fields/eyes from six eyes. *p < 0.05, **p < 0.01 (two-tailed unpaired *t*-test). **(C)** Immunostaining results showed that iNOS was elevated after hydrogen peroxide stimulation on cultured BV2 microglia, in particular expressed in the cytoplasm. In the AAT-treated microglia, not only the expression of iNOS significantly decreased, but also apparent upregulation of Arg1 expression was observed. Scare bar, 20 µm. **(D)** Western blot revealed that AAT downregulated the expression of iNOS while upregulated the Arg1 expression, the counteracting factor of iNOS.

The effect of AAT on the anti-inflammatory M2 microglia was also evaluated. CD206 and Arg1, two classic markers for M2 phenotype, were detected by immunofluorescence on retinal whole mounts and sections. We found significantly elevated ratio of CD206^+^IBA1^+^ microglia in retinal whole mounts from the AAT-treated group compared with the PBS-treated mice (Figure 7A). Similarly, although Arg1^+^IBA1^+^ M2 microglia were absent in the PBS-treated rd1 retinae, these M2 microglia appeared after AAT supplement (Figure 7B). From retinal sections, we found increased CD206^+^ and Arg1^+^ microglia locating mainly in the ganglion cell layer and ONL layers (Figures S1A,B in Supplementary Material). *In vitro*, immunostaining (Figure 7C) and western blot (Figure 7D) data revealed that AAT downregulated the expression of iNOS, another typical M1 marker, and upregulated Arg1 expression. CD206 also increased in the AAT-treated BV2 microglia (Figure S1C in Supplementary Material). These data suggested that AAT could induce microglia to adopt M2 phenotype.

### AAT Modulated IRF4/8 Activation and Phosphorylation of STAT1 in Microglia *In Vivo* and *In Vitro*

**Figure 8:**
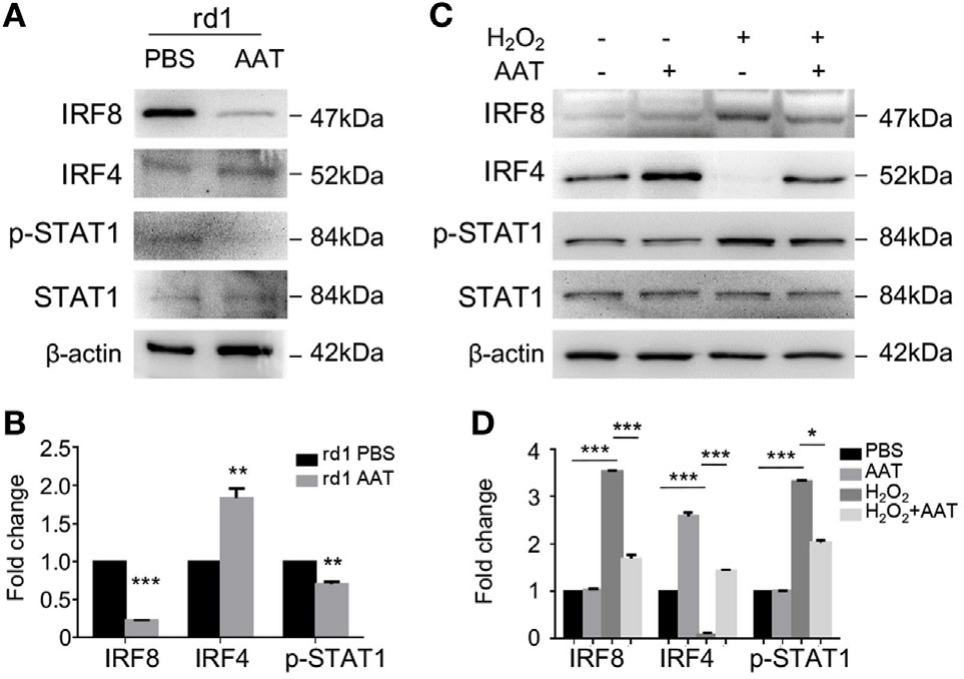
Alpha-1 antitrypsin (AAT) modulated IRF4/8 activation and phosphorylation of STAT1 *in vivo* and *in vitro*. **(A,B)** Western blot revealed that in AAT-treated retina of rd1 mice, the IRF8 expression significantly decreased while the expression of IRF4 increased slightly. Moreover, the STAT1 signaling was suppressed after AAT supplement. **(C,D)** *In vitro*, hydrogen peroxide stimulation upregulated the expression level of IRF8 and promoted phosphorylation of STAT1 in the primary cultured microglia, while AAT treatment reversed the expression trend with decreased IRF8 and STAT1 and increased IRF4. *n* = 3. *p < 0.05, **p < 0.01, ***p < 0.001 (two-tailed unpaired *t*-test).

The interferon-regulatory factor (IRF) members are transcriptional regulators of macrophage polarization, with IRF8 and IRF4 associated with the polarization toward the M1 state and M2 state, respectively (26, 27). We next detected the expressions of IRF4, IRF8, and relevant STAT1 signaling using western blot. The data revealed that in the AAT-treated retinae from rd1 mice, IRF8 expression was significantly suppressed while the level of IRF4 was slightly increased (Figures 8A,B). In addition, STAT1 signaling, of which phosphorylation could induce IRF8 expression, was also inactivated with the lack of p-STAT1 after AAT supplement (Figures 8A,B). Similarly, AAT stimulation downregulated the expression of IRF8 and upregulated IRF4 level in the cultured primary microglia. In addition, the phosphorylation of STAT1 was inhibited in the AAT-treated primary microglia cells (Figures 8C,D). Thus it is possibly that AAT exerted its modulatory effect through regulating the bias of IRF4 and IRF8 expression and STAT1 activation.

## Discussion

Neuroinflammation plays a key role in the process of RP (9, 10, 28). In the present study, the efficacy of AAT, an old drug showing new tricks of immunomodulatory property, was evaluated for preventing the neuroinflammation during the pathogenesis of retinal degeneration in rd1 mice. Our data provided evidence for the first time that AAT could suppress the neuroinflammation and attenuate neurodegeneration in the rd1 mice through the induction of anti-inflammatory M2 microglia, underlining that an immunomodulatory therapy may be an efficient strategy to retard retinal degeneration.

Alpha-1 antitrypsin, a 52-kDa glycoprotein encoded by the Serpina gene, is produced mainly by hepatocytes and maintained in circulation (22). Actually, the circulating AAT could be transported by vascular endothelial cells in an endocytosis pathway (29, 30). It is likely that AAT supplement by intraperitoneal injection enter retina through the blood–retinal barrier. Interestingly, AAT could be produced locally by mononuclear phagocytes in tissues and the expression is often reduced in a context of inflammation (21). For example, the amount of AAT in NOD diabetic islet was significantly less than that in C57BL/6 mice (12, 16, 17). In this study, we also found reduced expression of AAT in the rd1 retina, which was associated with neuroinflammation. So it is reasonable and promising of AAT supplement to treat RP due to the decreased level of AAT.

Accumulating evidence indicated that AAT exerted a novel immunomodulatory effect at the setting of inflammation. It is currently available and is used as medication in clinical trial for treating individuals with recent onset of type 1 diabetes and graft-versus-host disease, based on its potent immunomodulatory property (31, 32). Besides, AAT exhibits notable safety profile in clinical usage (33). Hence, it merits further investigation in other settings and our data of its efficacy in RP provide evidence to extend its usages in attenuating inflammation and protect the degenerative retina. In addition, it is well known that rd1 mice, an acute autosomal recessive form for human RP, carries a mutation affecting the expression of β subunit of phosphodiesterase 6, leading to the accumulation of cGMP that is thought to trigger photoreceptor degeneration in a short time. So these mice are hard to get complete recovery by intervention with biological agents. Despite the effect of immune therapy was not as remarkable as that of gene therapy in the rd1 mouse, suppression of neuroinflammation by AAT would provide a suitable microenvironment for cell survival and a stable treatment window for other therapies, considering the fact that neuroinflammation is associated with various type of RP, it serves as a hallmark of common pathological process of RP.

Alpha-1 antitrypsin exerted its immunomodulatory effect on many cellular targets (32, 34). Previous studies showed that neutrophils and monocyte/macrophage could produce more IL-10 and reduced the generation of TNF-α after AAT stimulation (35). AAT could also regulate the activities of NK cells through the interaction of NK cells and dendritic cells (DC) (34). It is worth noting that AAT modifies the phenotype of DC and B lymphocytes toward a tolerogenic pattern, exhibiting the immunomodulatory potential (36). In the present study, retinal microglia in rd1 mice experienced M2 polarization after AAT supplement, providing a novel immunological cellular mechanism by which AAT exerts its pluripotential anti-inflammatory effects. Despite the retina was believed to be immune privilege, its function was monitored by the active microglia, the main resident innate immune cells in CNS, who could initiate a cascade of cellular responses and orchestrate the immune condition in retinal diseases (10, 37). Similar to other immune cells like macrophages and DC, microglia adopt various phenotypes to adapt themselves to different insults (38). Our previous study has demonstrated that retinal microglia were activated and particularly polarized to a pro-inflammatory M1 phenotype at the rapid rods degenerative phase of rd1 mice (23), suggesting that M1 microglia contribute to retinal inflammation and the conversion to M2 anti-inflammatory statue seemed to be an efficient therapy for preventing retinal degeneration. Our findings suggested that AAT upregulated M2 anti-inflammatory activity and ameliorated the retinal degeneration, offering a novel immunological mechanism of AAT, particularly in the neurodegenerative diseases, in which microglia polarization is of importance. Of course, the effects of AAT on other retinal cell types could not be excluded in the rd1 mice, and it would be interesting to explore in future study.

A number of studies have implicated that transcription factors cooperatively regulated the downstream M1/M2-associated genes of macrophage/microglia (39). Among them, the IRF family could regulate maturation and activation of immune cells (40). IRF4 and IRF8 are two homologs in the IRF family whose expressions are largely restricted to lymphoid and myeloid cells. In retina, IRF4 and IRF8 were reported to be expressed mainly in microglia (26), which shared features with myeloid/hematopoietic lineage cells. Therefore, their expression and alteration in the whole retina were believed, to some extent, to be associated with microglial activities. IRF8 could induce the expressions of a large panel of M1-related genes, such as IFN-β, IL-12, iNOS, and so on (40, 41), whereas IRF4 functions as a negative regulator of TLRs signaling, associated with the polarization to M2 state (41, 42). In this study, high IRF8 and low IRF4 were observed in rd1 retina. *In vitro*, we found hydrogen peroxide insults induced a marked elevation of IRF8 and reduction of IRF4 in primary microglia, associating with more iNOS^+^ M1 and less ARG1^+^ M2 microglia, further indicating the critical role of IRF8 on M1 polarization of microglia. Strikingly, AAT altered the expression profile with lower IRF8 and higher IRF4 and induced microglia polarization toward M2 and away from M1 *in vivo* and *in vitro*, supporting the concept that AAT modulated microglia polarization partly through regulating the balance of IRF4 and IRF8 expression. A further understanding of mechanisms involved in microglia polarization and its regulation by AAT would facilitate the intervention of neuroinflammation, as well as improve and broaden the usage of AAT in neurodegenerative diseases.

In summary, we demonstrated in the present study that treatment with AAT attenuated neuroinflammation through shifting M1–M2 microglia polarization and alleviated retinal degeneration in rd1 mice. Furthermore, AAT regulated the expression level of IRF8 and IRF4, which were associated with reduced STAT1 phosphorylation, indicating that these signaling pathways were required in AAT-induced M2 polarization. Our data provide evidences of the anti-inflammatory and immunomodulatory properties of AAT and support the possibility that immunomodulatory therapy through regulating microglia M1/M2 polarization may be benefit for treating retinal degeneration.

## Ethics Statement

This study was approved by the animal experimental ethics committee of Zhongshan Ophthalmic Center, Sun Yat-sen University (authorized number: 2014-039). All the experiments were carried out in accordance with the approved guidelines of Animal Care and Use Committee of Zhongshan Ophthalmic Center and the Association Research in Vision and Ophthalmology (ARVO) Statement for the Use of Animals in Ophthalmic and Vision Research.

## Author Contributions

CH and XL designed and analyzed the experiments and wrote the manuscript. TZ, ZH, XZ, XS, YL, ML, and BC performed the experiments and analyzed the data. YL assisted in the experimental design and provided key research tools.

## Conflict of Interest Statement

The authors declare that the research was conducted in the absence of any commercial or financial relationships that could be construed as a potential conflict of interest.

## Supplementary Material

Figure S1 Alpha-1 antitrypsin (AAT) promoted the expression of M2 microglia markers. **(A,B)** In retinal section, CD206^+^IBA1^+^ microglia could hardly be found in PBS-treated group, and after AAT treatment, they prominently accumulated in the outer nuclear layer. The Arg1^+^IBA1^+^ microglia were also increased in AAT group. Scare bar, 20 µm. **(C)**. In the cultured microglia under oxidative stress, CD206 was rarely expressed with 200 μM hydrogen peroxide stimulation, while its expression significantly increased after AAT treatment, with more co-staining of CD206^+^ cells and IBA1. Scare bar, 20 µm.

## Abbreviations

AAT, alpha-1 antitrypsin; RP, retinitis pigmentosa; RPE, retinal pigment epithelium; ERG, electroretinogram; OCT, optical coherence tomography; GCL, ganglion cell layer; IPL, inner plexiform layer; INL, inner nuclear layer; OPL, outer plexiform layer; ONL, outer nuclear layer; CNS, central nervous system; IRF, interferon-regulatory factor.
