# Supplementary figures and images for: Alpha-1 Antitrypsin Attenuates M1 Microglia-Mediated Neuroinflammation in Retinal Degeneration
Source: Front Immunol. 2018 May 30;9:1202. doi: 10.3389/fimmu.2018.01202 (PMC5988858; doi:10.3389/fimmu.2018.01202)

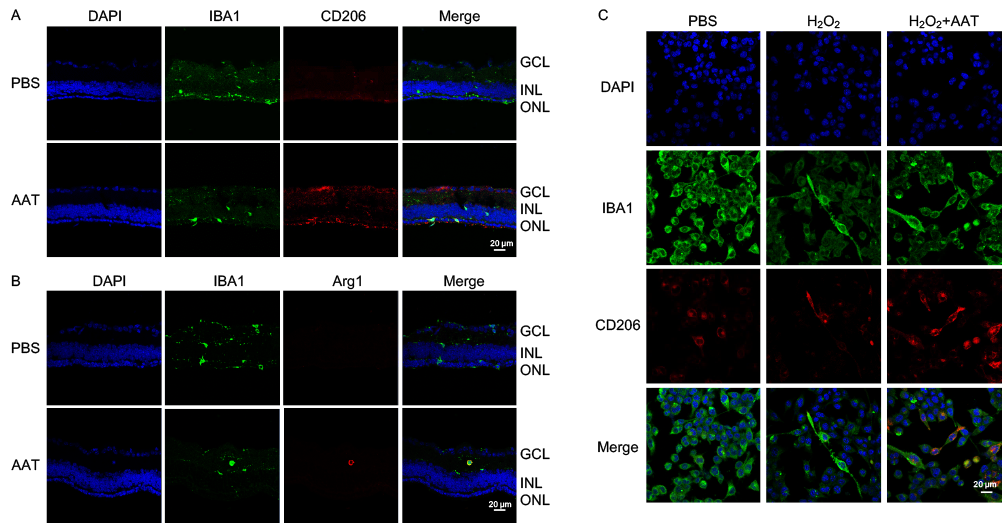

Supplement: Figure S1 — Alpha-1 antitrypsin (AAT) promoted the expression of M2 microglia markers. (A,B) In retinal section, CD206+IBA1+ microglia could hardly be found in PBS-treated group, and after AAT treatment, they prominently accumulated in the outer nuclear layer. The Arg1+IBA1+ microglia were also increased in AAT group. Scare bar, 20 µm. (C). In the cultured microglia under oxidative stress, CD206 was rarely expressed with 200 μM hydrogen peroxide stimulation, while its expression significantly increased after AAT treatment, with more co-staining of CD206+ cells and IBA1. Scare bar, 20 µm. [file Image_1.tif]
