# Supplementary material for: Alpha-1 Antitrypsin Attenuates M1 Microglia-Mediated Neuroinflammation in Retinal Degeneration
Source: Front Immunol. 2018 May 30;9:1202. doi: 10.3389/fimmu.2018.01202 (PMC5988858; doi:10.3389/fimmu.2018.01202)
Supplement: Supplementary file 2 [file Table_1.doc]

Table S1: Antibodies used in the study

| Abbreviates | Source | Dilution | Host | Application | References |
| --- | --- | --- | --- | --- | --- |
| AAT | Abcam  ab166610 | 1:100  1:1000 | Rabbit | IF  WB | (1) |
| CD11b | Abcam  ab8878 | 1:100 | Rat | IF | (2) |
| CD68 | Abcam  ab53444 | 1:100 | Rat | IF | (3) |
| IBA1 | Wako  Cat. No. 019-19741, | 1:100 | Rabbit | IF | (4) |
|  | Abcam  ab15690 | 1:100 | Mouse | IF |  |
| GFAP | Abcam  ab7260 | 1:100 | Rabbit | IF | (5) |
| Brn3a | Abcam  ab81213 | 1:100 | Rabbit | IF | (6) |
| CD16/32 | BD Biosciences  553141 | 1:100 | Rat | IF | (7) |
| iNOS | Santa Cruz  sc-7271 | 1:50  1:500 | Mouse | IF  WB | (8) |
| CD206 | R&D Systems  AF2535 | 1:100 | Mouse | IF | (9) |
| Arg1 | Santa Cruz  sc-18355 | 1:50  1:500 | Goat | IF  WB | (10) |
| Rhodopsin | Santa Cruz  sc-57432 | 1:50 | Mouse | IF | (11) |
| IRF4 | Thermo Fisher Scientific  PA5-21144 | 1:1000 | Rabbit | WB | (12) |
| IRF8 | Abcam  ab28696 | 1:1000 | Rabbit | WB | (13) |
| STAT1 | CST  14994S | 1:1000 | Rabbit | WB | (14) |
| P-STAT1 | CST  7649S | 1:1000 | Rabbit | WB |  |
| β-actin | Abcam  ab-28696 | 1:2000 | Mouse | WB | (15) |

References:

1. du Bois RM, Bernaudin JF, Paakko P, Hubbard R, Takahashi H, Ferrans V, et al. Human neutrophils express the alpha 1-antitrypsin gene and produce alpha 1-antitrypsin. *Blood* (1991) **77**(12):2724-30.

2. Roy A, Fung YK, Liu X, Pahan K. Up-regulation of microglial CD11b expression by nitric oxide. *The Journal of biological chemistry* (2006) **281**(21):14971-80. doi: 10.1074/jbc.M600236200.

3. Kobayashi K, Imagama S, Ohgomori T, Hirano K, Uchimura K, Sakamoto K, et al. Minocycline selectively inhibits M1 polarization of microglia. *Cell death & disease* (2013) **4**:e525. doi: 10.1038/cddis.2013.54.

4. Le Blon D, Hoornaert C, Daans J, Santermans E, Hens N, Goossens H, et al. Distinct spatial distribution of microglia and macrophages following mesenchymal stem cell implantation in mouse brain. *Immunology and cell biology* (2014) **92**(8):650-8. doi: 10.1038/icb.2014.49.

5. Dai J, Fu Y, Zeng Y, Li S, Qin Yin Z. Improved retinal function in RCS rats after suppressing the over-activation of mGluR5. *Scientific reports* (2017) **7**(1):3546. doi: 10.1038/s41598-017-03702-z.

6. Cerman E, Akkoc T, Eraslan M, Sahin O, Ozkara S, Vardar Aker F, et al. Retinal Electrophysiological Effects of Intravitreal Bone Marrow Derived Mesenchymal Stem Cells in Streptozotocin Induced Diabetic Rats. *PloS one* (2016) **11**(6):e0156495. doi: 10.1371/journal.pone.0156495.

7. Higashi Y, Aratake T, Shimizu S, Shimizu T, Nakamura K, Tsuda M, et al. Influence of extracellular zinc on M1 microglial activation. *Scientific reports* (2017) **7**:43778. doi: 10.1038/srep43778.

8. Shen X, Burguillos MA, Osman AM, Frijhoff J, Carrillo-Jimenez A, Kanatani S, et al. Glioma-induced inhibition of caspase-3 in microglia promotes a tumor-supportive phenotype. *Nature immunology* (2016) **17**(11):1282-90. doi: 10.1038/ni.3545.

9. Azad AK, Rajaram MV, Schlesinger LS. Exploitation of the Macrophage Mannose Receptor (CD206) in Infectious Disease Diagnostics and Therapeutics. *Journal of cytology & molecular biology* (2014) **1**(1). doi: 10.13188/2325-4653.1000003.

10. Sindrilaru A, Peters T, Wieschalka S, Baican C, Baican A, Peter H, et al. An unrestrained proinflammatory M1 macrophage population induced by iron impairs wound healing in humans and mice. *The Journal of clinical investigation* (2011) **121**(3):985-97. doi: 10.1172/JCI44490.

11. Di Pierdomenico J, Garcia-Ayuso D, Pinilla I, Cuenca N, Vidal-Sanz M, Agudo-Barriuso M, et al. Early Events in Retinal Degeneration Caused by Rhodopsin Mutation or Pigment Epithelium Malfunction: Differences and Similarities. *Frontiers in neuroanatomy* (2017) **11**:14. doi: 10.3389/fnana.2017.00014.

12. Zhang Y, Jia X, Xia Y, Li H, Chen F, Zhu J, et al. Altered expression of transcription factors IRF4 and IRF8 in peripheral blood B cells is associated with clinical severity and circulating plasma cells frequency in patients with myasthenia gravis. *Autoimmunity* (2018):1-9. doi: 10.1080/08916934.2018.1454913.

13. McEwan WA, Tam JC, Watkinson RE, Bidgood SR, Mallery DL, James LC. Intracellular antibody-bound pathogens stimulate immune signaling via the Fc receptor TRIM21. *Nature immunology* (2013) **14**(4):327-36. doi: 10.1038/ni.2548.

14. De Wilde K, Martens A, Lambrecht S, Jacques P, Drennan MB, Debusschere K, et al. A20 inhibition of STAT1 expression in myeloid cells: a novel endogenous regulatory mechanism preventing development of enthesitis. *Annals of the rheumatic diseases* (2017) **76**(3):585-92. doi: 10.1136/annrheumdis-2016-209454.

15. Chen J, Li Z, Hatcher JT, Chen QH, Chen L, Wurster RD, et al. Deletion of TRPC6 Attenuates NMDA Receptor-Mediated Ca(2+) Entry and Ca(2+)-Induced Neurotoxicity Following Cerebral Ischemia and Oxygen-Glucose Deprivation. *Frontiers in neuroscience* (2017) **11**:138. doi: 10.3389/fnins.2017.00138.
